# Chronic kidney disease, severe arterial and arteriolar sclerosis and kidney neoplasia: on the spectrum of kidney involvement in MELAS syndrome

**DOI:** 10.1186/1471-2369-13-9

**Published:** 2012-02-21

**Authors:** Giorgina Barbara Piccoli, Laura Davico Bonino, Paola Campisi, Federica Neve Vigotti, Martina Ferraresi, Federica Fassio, Isabelle Brocheriou, Francesco Porpiglia, Gabriella Restagno

**Affiliations:** 1 SS Nephrology ASOU san Luigi, Orbassano, Torino, Italy; 2 Anatomia Patologica ASOU Molinette, Torino, Italy; 3 Department of Pathology, Laboratory of Pathology, Molinette Hospital, Turin, Italy; 4 Assistance Publique- Hôpitaux de Paris, Hôpital Tenon, Department of Pathology, Université Pierre et Marie Curie Univ Paris 06, Paris, France; 5 Genetica Medica, ASOU OIRM sant'Anna, Torino, Italy; 6 Department of Medical and Biological Sciences. SS Nephrology, ASOU san Luigi Gonzaga, Regione Gonzole, Orbassano, Torino, Italy

**Keywords:** MELAS syndrome, Chronic kidney disease, Renal vascular, Disease, Kidney cancer

## Abstract

**Background:**

MELAS syndrome (MIM ID#540000), an acronym for Mitochondrial Encephalopathy, Lactic Acidosis and Stroke-like episodes, is a genetically heterogeneous mitochondrial disorder with protean manifestations and occasional kidney involvement. Interest in the latter is rising due to the identification of cases with predominant kidney involvement and to the hypothesis of a link between mitochondrial DNA and kidney neoplasia.

**Case presentation:**

We report the case of a 41-year-old male with full blown MELAS syndrome, with lactic acidosis and neurological impairment, affected by the "classic" 3243A > G mutation of mitochondrial DNA, with kidney cancer. After unilateral nephrectomy, he rapidly developed severe kidney functional impairment, with nephrotic proteinuria. Analysis of the kidney tissue at a distance from the two tumor lesions, sampled at the time of nephrectomy was performed in the context of normal blood pressure, recent onset of diabetes and before the appearance of proteinuria. The morphological examination revealed a widespread interstitial fibrosis with dense inflammatory infiltrate and tubular atrophy, mostly with thyroidization pattern. Vascular lesions were prominent: large vessels displayed marked intimal fibrosis and arterioles had hyaline deposits typical of hyaline arteriolosclerosis. These severe vascular lesions explained the different glomerular alterations including ischemic and obsolescent glomeruli, as is commonly observed in the so-called "benign" arteriolonephrosclerosis. Some rare glomeruli showed focal segmental glomerulosclerosis; as the patient subsequently developed nephrotic syndrome, these lesions suggest that silent ischemic changes may result in the development of focal segmental glomerulosclerosis secondary to nephron loss.

**Conclusions:**

Nephron loss may trigger glomerular sclerosis, at least in some cases of MELAS-related nephropathy. Thus the incidence of kidney disease in the "survivors" of MELAS syndrome may increase as the support therapy of these patients improves.

## Background

MELAS syndrome (MIM ID#540000), an acronym for Mitochondrial Encephalopathy, Lactic Acidosis and Stroke-like episodes, is a genetically heterogeneous mitochondrial disorder with protean manifestations, different expressivity and occasional kidney involvement [1-5]. The multisystem presentation, variable onset and severe cerebrovascular involvement are exemplified by the history of the philosopher and poet Friedrich Nietzsche who died at the age of 55 after a mysterious illness. Whether or not the MELAS diagnosis is true, this historical note has the merit of highlighting the variable manifestations and clinical mimicry of this rare disease [6].

MELAS syndrome is associated in about 80% of cases with a mutation at position 3243 in the mitochondrial gene MTTL1 (MIM ID *590050), encoding mitochondrial tRNA leucine 1(3243A > G transition), although other mutations have also been described [7]. Interest in kidney involvement is rising due to the identification of MELAS mutations in cases with predominant kidney involvement, occasionally with very late diagnosis, and to the description of a putative link between mitochondrial DNA and different types of kidney neoplasia [8-12].

Kidney involvement is protean, and both glomerular disease (focal segmental glomerulosclerosis- FSGS) and tubular lesions, resulting in hyponatremia or in a full-blown De Toni Fanconi Debré syndrome, have been described. Furthermore, in several of the reported cases, kidney involvement was diagnosed only when advanced renal failure occurred [13-19]. The best-characterized lesion, leading to kidney biopsy in nephrotic syndrome, is FSGS. The role of early ischemic changes in the reduction of the nephron population has not yet been elucidated [13-19].

We report the case of a patient with MELAS syndrome and the "classic" 3243A > G mutation of mitochondrial DNA, who developed kidney cancer and severe and rapid kidney functional impairment after unilateral nephrectomy. Analysis of the kidney tissue at a distance from the cancer lesion, sampled in an early phase of kidney disease, in the context of normal blood pressure, recent onset of diabetes and before the appearance of proteinuria, may allow a better understanding of the pathogenesis of the renal lesions in MELAS syndrome.

## Case presentation

A 41-year-old male was referred to our Nephrology Unit because of worsening of kidney function and proteinuria. He was born at term of a normal pregnancy from apparently healthy, nonconsanguineous parents. The patient's early development was normal and he had no evident clinical problem until 18 years of age, when he developed seizures triggered by light and recurrent headaches. He was prescribed Valproic Acid and started a discontinuous follow-up in Neurology. In 1991 (age 22) EEG revealed well-represented alpha background activity, with a complex wave-peak after 7-10 flashes/sec, an expression of an epileptogenic photosensitive centroencephalic focus. In 1992, in the presence of signs of myopathy (asthenia, increase of serum lactate, myalgia), a muscle biopsy showed non-specific changes (occasional small red sub-sarcolemmal deposits, showing oxidative enzymatic activity). In 1992 the patient's sister (3 years younger) developed generalized seizures and myopathy. Genetic counseling suggested the presence of a mitochondrial disease.

Detection of the 3243A > G mutation of mitochondrial DNA confirmed the diagnosis of MELAS syndrome in the patient, his sister, his mother and one of the mother's sisters.

In the subsequent decade, he started and discontinued different types of anticonvulsant therapy (including Lamotrigine and Phenobarbital), and developed recurrent seizures, with imaging evidence of an ischemic lesion in the left hemisphere, progressive bilateral sensorineural hearing loss and sensitive distal polyneuropathy with increasing muscle weakness. On various occasions, lactic acidosis was found (lactic acid 10-13.5 mmol/L; normal <2 mmol/L).

In July 2009 (age 39) he was diagnosed with diabetes mellitus (weight loss, polyuria and polydipsia); at diagnosis, glycated hemoglobin was 19.6% and glycemia <500 mg/dL. Insulin treatment was started. Soon after diagnosis, he developed a sepsis due to *Klebsiella pneumoniae*. A CT scan, performed on the suspicion of a pyelonephritis focus, showed a solid hypervascularized mass, highly suggestive of neoplasia, in the right kidney. During hospitalization, serum creatinine was 0.9 mg/dL; 24-hour proteinuria was absent at repeated urinalysis; glomerular filtration rate (eGFR) was 62 mL/min (Cockcroft and Gault formula). He was normotensive (usual blood pressure 120/80 mmHg) and remained so during follow-up.

**Figure 1:**
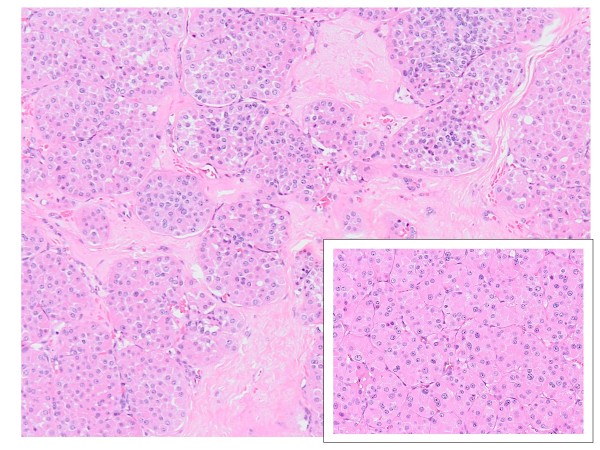
**Renal oncocytoma: the tumor consists of solid, compact nests, acini, tubules or microcysts within a hypocellular hyalinized stroma**. **(a)**. In the box: At high-power magnification the predominant cell type, the so-called oncocyte, is round to polygonal with granular eosinophilic cytoplasm, round regular nuclei with evenly dispersed chromatin and a centrally placed nucleolus **(b) **(Hematoxylin eosin stain)

**Figure 2:**
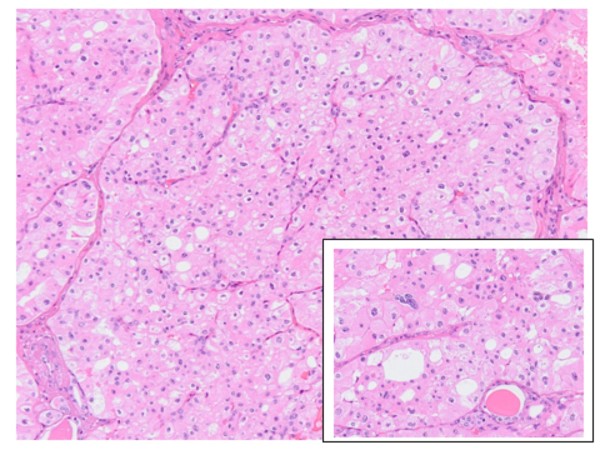
**The smaller lesion (0.5 cm): the figure shows a solid growth pattern and perinuclear cytoplasmic clearing**. **(a)**. In the box: The tumor contains a population of large, round to polygonal cells with well-defined cell borders and amphophilic, pale basophilic to foamy cytoplasm; nuclei are typically hyperchromatic, elongated and grooved with an irregular nuclear membrane. These features suggest chromophobe renal cell carcinoma; alternatively the lesion may represent a focus of tubular oncocytosis and a precursor of another oncocytoma; the two types of lesions are in any case strictly correlated [43,44]**(b) **(Hematoxylin eosin stain)

In November 2009 the patient underwent right nephrectomy. At surgery, a second small superficial tumor was detected. The histological examination identified a renal oncocytoma (2 cm); a smaller lesion (0.5 cm) had an appearance highly suggestive of chromophobe carcinoma. However, the lesion could also represent a tumoral focus of tubular oncocytosis and a precursor of another oncocytoma (Figures 1 and 2). The cytogenetic analysis was not performed, in this case; however, the two lesions are highly correlated, and a further definition was considered as of minor clinical relevance.

At hospital discharge, serum creatinine was 1.17 mg/dL with mild proteinuria (30 mg/dL). In the same year the patient's sister developed breast cancer and was identified as a carrier of the BRCA-2 mutation. However, the other family members, including our patient, preferred not to perform genetic testing for the BRCA mutation.

In January 2011 the patient was referred to our center. Glycemic control was good (glycated hemoglobin 6.5% on Repaglinide). Blood pressure was normal, without therapy. Cognitive impairment and psychomotor agitation, choreiform movements and intentional tremor, nystagmus and lower limb muscle weakness, hypotrophy and severe muscle wasting were present (height 155 cm, weight 36 Kg, BMI: 15). His speech was slow, slurred but coherent, and he appeared severely depressed. His last biochemical tests were: serum creatinine 2.1 mg/dL (eGFR 23 mL/min), proteinuria 3.6 g/24 hours, low-normal total proteins (6.2 g/dL) and normal serum albumin (3.7 g/dL). EKG was normal; no sign of cardiomyopathy was detected. Further tests showed: low IgG levels (499 mg/dL), normal IgA and IgM (213 and 115 mg/dL respectively); high PTH (445 pg/mL); moderate acidosis (pH 7.227; HCO3 22.8; SBE -3.7; normal lactates: 1.2 mmol/L); antinuclear antibodies and ANCA were negative.

Renal ultrasounds showed moderate medullary hyperechogeneity, consistent with tubulo-interstitial or vascular damage.

The rapid progression of kidney disease, with the development of nephrotic proteinuria, led us to review the samples of kidney tissue at a distance from the cancer lesions.

Of note, the changes were recorded before the onset of proteinuria and in the absence of hypertension, both at nephrectomy and over the follow-up.

**Figure 3:**
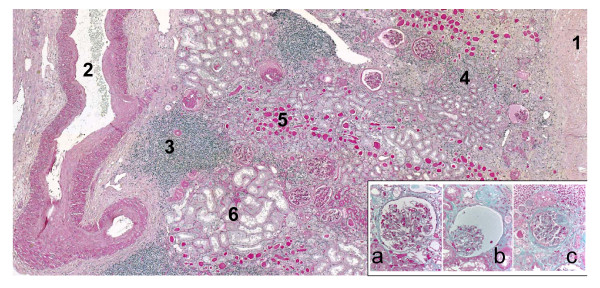
**Overview of cortical atrophy: the thickness of the renal parenchyma is severely reduced (only 3 mm between the renal capsule (1) and the arcuate artery (2))**. There is widespread interstitial fibrosis, with dense inflammatory infiltrate (**3**), some tubules are atrophic with either the classical pattern (**4**) or with thyroidization type (**5**), some residual tubules are hypertrophic (**6**). The arcuate artery shows a severe narrowing of the lumen by marked intimal fibrosis (PAS stain). In the box: The figure shows the different patterns of glomeruli: normal (**a**), ischemic or obsolescent (**b**), focal segmental glomerulosclerosis (**c**) (Masson trichromatic stain)

**Figure 4:**
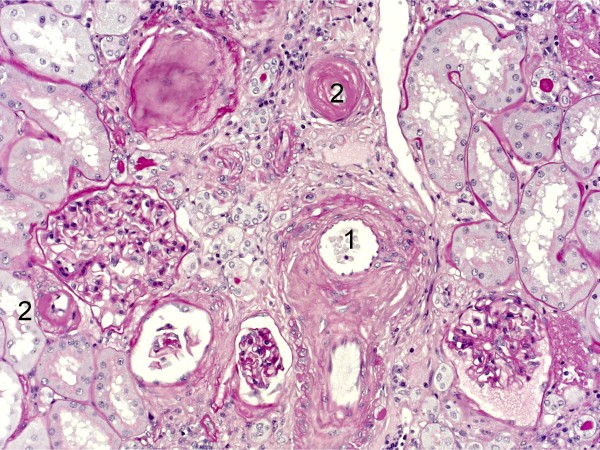
**Severe impairment of an artery with marked intimal fibrosis leading to narrowing of the lumen**. **(1)**. Small arteries (**2**) have hyaline deposits (PAS stain)

Morphological examination revealed a widespread interstitial fibrosis and marked vascular changes characterized by intimal fibrosis of arcuate and interlobular arteries with a wedge-shaped area of interstitial fibrosis and tubular atrophy extending to the renal capsule suggestive of ischemic damage (Figure 3). Hyaline arteriolosclerosis (Figures 3 and 4) and signs of chronic inflammation were also seen. These severe vascular lesions were the basis of the different glomerular alterations including ischemic and obsolescent glomeruli. The obsolescent glomeruli amounted to approximately 50% of the more than 100 glomeruli sampled. Some rare glomeruli (about 5-10%) showed focal segmental glomerulosclerosis, which in this context can be interpreted as consequent to the vascular alterations.

As the patient subsequently developed nephrotic syndrome, these lesions suggest that silent ischemic changes may result in the development of focal segmental glomerulosclerosis secondary to nephron loss, at least in some cases of MELAS-related nephropathy.

## Conclusions

The present report regards an adult patient presenting all the clinical criteria for MELAS syndrome (stroke-like episodes, with onset before age 40; encephalopathy with seizures and cognitive impairment; mitochondrial myopathy with lactic acidosis, in the context of normal early psychomotor development and recurrent headache), as well as the most frequent complications, namely diabetes mellitus (initially defined as type 2 diabetes, but at present more often defined as mitochondrial diabetes) and sensorineural hearing loss [1]. Molecular genetic testing identified the most common mutation found in MELAS patients, MTTL1 (MIM ID *590050), encoding mitochondrial tRNA leucine 1(3243A > G transition) [1,20,21].

The feature of this case is the severity and complexity of kidney involvement, characterized by kidney cancer and by severe arteriolonephrosclerosis occurring in the absence of hypertension, which we speculate may be the basis for the severe and progressive nephropathy (with the appearance of nephrotic proteinuria, previously absent) that developed shortly after nephrectomy (Figures 1 and 4).

Kidney involvement is reported to be rare and is protean in MELAS patients [1,20]. Reported cases include: glomerular diseases, mainly FSGS; tubular disorders, either with Fanconi syndrome or in the form of salt-losing nephropathies; end-stage renal failure [2-4,10-19,21-23]. The presence of abnormal mitochondria in tubules and in podocytes has been described since the first reports, suggesting a direct role of mitochondrial alterations in the pathogenesis of tubular derangements and of steroid-resistant focal segmental glomerulosclerosis; unfortunately electron microscopy was not available in our case, thus impairing any conclusion on the podocyte status in our patient [12,19]. Most cases of kidney involvement are described in adolescent and adult patients [2-4,7,8,11-19]. This suggests that kidney disease may develop in the "survivors" of MELAS syndrome; if this proves to be true, its incidence may increase as the support therapy improves [22]. To our knowledge, the role of ischemic changes, eventually resulting in the development of glomerulosclerosis secondary to nephron loss and nephrotic range proteinuria, has never been clearly described, also because these early changes may be greatly underestimated as they are typically neither symptomatic nor detected by common laboratory tests until more than 50% of the kidney parenchyma is damaged.

Our patient was normotensive and non-proteinuric at the time of nephrectomy, even if his renal function had probably started to decrease. Indeed, the assessment of glomerular filtration rate may be a challenge in MELAS syndrome: none of the commonly employed formulae for GFR assessment is precise in the presence of muscle wasting. We chose the Cockcroft and Gault formula as it takes into account the patient's weight; however, neither this nor the MDRD or EPI formulae are reliable with very low BMI (15 in our patient) and 24-hour urine studies may be biased by higher creatinine excretion due to myopathy [24].

The kidney tissue analysis showed a severe vascular nephropathy with ischemic damage affecting glomeruli despite the absence of hypertension (Figure 3). After nephrectomy, in parallel with the attainment of very good diabetes control, he progressively developed nephrotic proteinuria and kidney functional impairment, whose kinetics (a few months after nephrectomy) suggested an effect similar to 5/6 nephrectomy [25-27]. Thus, a non-immunologic, non-primarily metabolic but hemodynamic mechanism of increased work-load on the remnant nephrons probably contributed to the development of FSGS in our patient. Of course, the possibility of another nephropathy different from FSGS cannot be completely ruled out, although this is highly unlikely given the disease course and the genetic background.

Our data do not contrast with the reports of altered mitochondria in several kidney compartments, being analogous to what has been described in the brain, where ischemic lesions coexisted with diffuse cellular alterations and where swollen and altered mitochondria were found in different cell types, including neuronal and endothelial cells [1,12,19,28-30]. The lack of steroid sensitivity of the nephrotic syndrome in MELAS patients may be due to the fact that the lesions are caused by mitochondrial alterations and by nephron loss of vascular origin [25-32].

The second interesting aspect of the kidney involvement in our patient is the presence of kidney neoplasia (Figures 2 and 3). As he refused to undergo further genetic testing, the role of the BRCA2 mutation could not be assessed. However, a relationship between kidney cancer and the BRCA2 mutation has not been reported thus far. Mitochondrial alterations have been linked to the development of cancer [33]. While there are some reports of an association with gallbladder, colon or thyroid cancers, very few reports have linked MELAS syndrome and kidney neoplasia; an exception was a clear renal cell carcinoma in a child, a type of renal cancer quite unusual in childhood [8,34-36]. Indeed, renal oncocytomas and chromophobe renal carcinomas, both derived from intercalated cells of the collecting duct, display morphological and functional alterations of the mitochondria [37-40]. A genetic analysis was not performed in our patient to further clarify the diagnosis; however, cromophobe carcinomas, oncocytomas and tubular oncocytosis are thought to be pathogenetically related and further definition of the lesions was not considered relevant to the case report.

Previous studies have shown that accumulation of mitochondria in renal oncocytomas is associated with somatic mutations of mitochondrial DNA, resulting in decreased activity of the respiratory chain complex [41], while heteroplasmic mitochondrial DNA mutations are found in chromophobe renal cell carcinomas [42]. Therefore, it is plausible that the mitochondrial DNA mutation detected in our patient was responsible for the development of the closely related cancers [43,44]. Moreover, oncocytoma may be the result of the intrinsic mitochondrial mutation combined with the tendency for neoplasms, including oncocytomas, to develop in chronically diseased kidneys. The occurrence of these rare and slowly progressive kidney cancers in MELAS syndrome should be kept in mind in view of the importance of nephronsparing policies in patients at risk of end-stage kidney disease because of progressive reduction of the nephron mass; due to their slow progression, their prevalence might be underestimated in patients with MELAS syndrome [37-40].

In conclusion, this case broadens the spectrum of kidney involvement in MELAS syndrome and may clarify some aspects of its pathogenesis. In particular, it shows that kidney vascular involvement may precede the development of nephrotic range proteinuria, suggesting that in some patients with MELAS syndrome the glomerular disease may be linked to the progressive reduction of the nephron mass. The association with kidney cancer suggests strict surveillance of MELAS patients for kidney neoplasia, while the rapid progression of kidney disease after nephrectomy underlines the importance of nephron-sparing policies in patients in whom renal function involvement may be "masked" by reduced muscle mass leading to "normal" creatinine values.

## Consent

The patient had given informed consent for the genetic analysis of MELAS syndrome at the time of genetic counseling. He gave informed consent to the publication of the present case report and other anonymous reports on his clinical history. He did not want to perform further genetic testing for the BRCA2 mutation, as he was convinced of his poor prognosis for vascular reasons.

A copy of the written consent is available for review by the Editor-in-Chief of this journal.

## Competing interests

The authors declare that they have no competing interests.

## Authors' contributions

GBP designed the study and drafted the manuscript, DBL and CP performed the analysis of the kidney neoplasia and participated in the discussion of the results; VFN was in charge of the clinical follow-up of the patient, FM performed the systematic literature review; GR and FF performed the genetic analysis, FP reviewed the urological aspects. BI reviewed the histology of the non-neoplastic areas of the kidney. All authors read and approved the final manuscript.

## Pre-publication history

The pre-publication history for this paper can be accessed here:

http://www.biomedcentral.com/1471-2369/13/9/prepub
